# Study on the browning mechanism of betel nut (*Betel catechu* L.) kernel

**DOI:** 10.1002/fsn3.1456

**Published:** 2020-03-10

**Authors:** Yuting Guo, Yonggui Pan, Zhengke Zhang, Weimin Zhang

**Affiliations:** ^1^ College of Food Science and Engineering Hainan University Haikou China

**Keywords:** betel nut kernel, enzymatic browning, O_2_, phenolic compounds, PPO

## Abstract

Taking betel nut (*Betel catechu* L.) kernel as raw materials, to analyze the browning mechanism of betel nut kernel. First, we extract and separate the phenolic substances and polyphenol oxidase (PPO) from the betel nut kernel and then find out how O_2_ penetrates into the kernel, study the above 3 key factors of enzymatic browning so as to prove the possibility of enzymatic browning, and further study the browning products to clarify the mechanism of browning of betel nut kernel during storage process. The results showed that 11 kinds of phenolic compounds were isolated and identified from the betel nut kernel by liquid chromatography–mass spectrometry, among which chlorogenic acid was the highest, followed by dopamine and L‐epicatechin, and other contents were relatively low. Meanwhile, PPO was also separated from the betel nut kernel by DEAE–Sepharose Fast Flow and Phenyl–Sepharose 6 Fast Flow column chromatography. On this basis, scanning electron microscopy showed that the damage of the betel nut tissue was aggravated with the prolonged storage time, the wax was gradually decomposed, and the stratum corneum of the peel is destroyed in a honeycomb‐shaped; the lignification of the flesh was aggravated, and the interstitial space was increased; and the crack of the kernel membrane was also enlarged. These changes of structure contribute to increase gas exchange within and outside the organization, including the entry of O_2_. Finally, the oxidation products generated from simulated reaction of chlorogenic acid, dopamine, and epicatechin with purified PPO under aerobic conditions in vitro were compared with the products extracted from naturally brown betel nut, and the same absorption spectra were found. Therefore, it indicates that the browning of betel nut kernel is an enzymatic browning caused by the reaction of phenolic substrates were oxidized by PPO.

## Graphical abstract

The oxidation products generated from simulated reaction of chlorogenic acid, dopamine, and epicatechin with purified polyphenol oxidase (PPO) under aerobic conditions in vitro were compared with the products extracted from naturally brown betel nut, and the same absorption spectra were found. It indicates that the browning of betel nut kernel is an enzymatic browning caused by the reaction of phenolic substrates were oxidized by PPO.

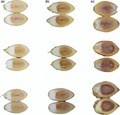

## 1 INTRODUCTION

Betel nut (*Betel catechu* L.) is the fruit of the Betel catechu tree. Approximately 700 million individuals regularly chew betel nut worldwide, which is one of the world's three largest oral addicting food (betel nut, chewing gum, cigarette), the fruit contains a variety of beneficial substances and nutrients required by the human body and has various functions such as refreshing and relieving pain and helping digestion (Peng, Liu, & Wu, 2015). In China, many consumers prefer to eat fresh betel nut because it not only maintains the original nutritional quality of betel nut but also reduces the damage to the mouth caused by dry betel nut fiber and its benzopyrene and other harmful substances (Li, Zhang, & Pan, 2017). However, the fresh betel nut is highly prone to browning of kernel during storage, which affects the fruit quality. Therefore, preservation of betel nut fruit has received much attention.

For most of fruits, browning generally occurs on the surface or injured parts (Toivonen & Brummell, 2008), while browning is rare in the interior of the fruit. Mainly because in the intact fruit, O_2_ absorbed by the main metabolic pathways can only maintain its normal respiration; excess O_2_ is excluded from the tissue, so that the tissue is isolated from oxygen and therefore cannot directly act on the enzyme and substrate to participate in the browning reaction (Sun et al., 2002). At present, research on the internal browning of intact fruits is mainly found in pears and pineapples, which are generally considered to be the result of enzymatic browning (Franck, Lammertyn, & Ho, 2007; Koushesh & Moradi, 2016; Luengwilai, Beckles, & Siriphanich, 2016). However, for the occurrence of enzymatic browning, three factors must be met: phenolic substances, polyphenol oxidase (PPO), and O_2_ (Adams & Brown, 2007; He & Luo, 2007; Mayer, 2006).

Up until now, there has been little research on the browning of betel nut, and there has been little research from its mechanism to control. However, antioxidant studies on betel nut showed that there are higher phenolic components in betel nut kernel (And & Lee, 2009; Han, Zhang, & Li, 2010; Zhang, Huang, & Chen, 2014), and this finding also implies the possibility of enzymatic browning in the betel nut kernel. However, from the perspective internal browning of pears and pineapples, combined with the presence of phenolic substances in betel nut kernel, we speculate that the mechanism should be the same as the result of enzymatic browning to produce melanin. This must be supported by evidence, including the phenol compounds—the substrates of the enzymatic browning, PPO—the key enzyme of enzymatic browning, and O_2_ in kernel, and there is a key question to study: how O_2_ enters the inside of the fruit tissue. Under normal circumstances, for O_2_ transport mechanism, three kinds of barriers to gas flow have to be considered: one of them is the peel, through which a gas is transported by a permeation mechanism that is affected, mainly, by the fruit cuticle structure and waxes deposited on it (Banks, Cleland, & Cameron, 1995; Amarante & Banks, 2001; Lammertyn, Scheerlinck, & Verlinden, 2001; Valle‐Guadarrama et al., 2002); the other one is the flesh of the fruit, where the transport mechanism occurs by of pore spaces diffusion (Lammertyn, Scheerlinck, Jancsók, Verlinden, & Nicolaï, 2003; Lammertyn et al., 2001; Zhang & Bunn, 2000); finally, the O_2_ diffuses within the tissue membrane to the point of O_2_ consumption. Therefore, it is necessary to study the possibility of O_2_ entering the kernel to participate in enzymatic browning from the histomorphology. In this research, the browning problem of the betel nut kernel during the storage process was studied from the perspective of enzymatic browning, so as to explore the mechanism of browning. Consequently, it provides a theoretical basis for the control of fresh betel nut browning, and it has important theoretical and practical significance to solve browning in the process of betel nut preservation.

## 2 MATERIALS AND METHODS

### 2.1 Fruit materials and treatments

Betel nut fruit was obtained from a commercial orchard in Hainan Province in China. Fruit was transported to the laboratory within 1h. Fruit with uniformity of color, size, and maturity, free mechanical injury was selected for experiment.

### 2.2 Experimental methods

#### 2.2.1 Extraction, separation, and identification of phenolic compounds from betel nut kernel

##### Extraction of betel nut kernel phenolic compounds

The Phenols were extracted according to the method reported previously by Pan (2006), with a slight modification. Fourty g betel nut kernel was grounded with 400 ml of precooled 65% ethanol containing 0.5% sodium bisulfite using a tissue grinder (DS‐1, Jingke Experiment Co., Ltd); then, the mixture was immersed in an ice bath for 30 min and filtered, the filtrate was combined and centrifuged (TGL‐16M, Luxiangyi Centrifuge Instrument Co., Ltd) at 12,875 *g* for 20 min, and the supernatant was vacuum evaporated (RE‐2000A, Yarong Biochemical Instrument Factory) at 50°C to remove the ethanol; then, petroleum ether was added to the residue in a ratio of 2:1 to remove the pigment, and subsequently, the organic phase was discarded; 20% ammonium sulfate and 2% metaphosphoric acid were added to the aqueous phase to remove the protein, then add an equal volume of ethyl acetate to repeatedly extract the phenolic substance twice; the ethyl acetate phase was collected, evaporated to dryness on a vacuum rotary evaporator at 30°C, the residue was dissolved in 10 ml of methanol, and the solution was centrifuged at 10,000 rpm for 10 min; the supernatant was filtered through a 0.22 μm microporous membrane（Jinteng Experimental Equipment Co., Ltd）to obtain a preliminarily purified phenolic material concentrate stored at −20°C until to use.

##### High‐performance liquid chromatography identification

The identification of HPLC was modified with reference to Rubio‐Senent et al. (2015). The phenolic compounds were identified using an Aglient 1100 liquid chromatography system with an Eclipse XDB‐C18 column (150 mm × 4.6 mm × 5 μm). The system was equipped with a UV detector, and the detection wavelength used for identification was 280 nm. Separation of the phenolic compounds extracts from betel nut kernel was carried out at a flow rate of 0.7 ml/min using a mixture water (H_2_O) acidified with 0.01% acetic acid (solvent A) and pure acetonitrile (CH_3_CN) (solvent B) as mobile phase, utilizing the following gradient over a total run time of 25 min: 0 min 90% A and 10% B; 25 min 85% A and 15% B, column temperature 30°C, injection volume 10 μl. According to the peak time, retention time, and peak shape, phenolic compounds were characterized.

##### Mass spectrometry detection conditions

Mass spectrometry was performed according to the method of Sahu et al. (2018). The effluent from the column in the HPLC was introduced into a triple quadrupole (Waters Xevo TQ‐S Micro) mass spectrometer detector, equipped with an electrospray ionization (ESI) interface. A split postcolumn of 0.5 ml/min was introduced directly on the mass spectrometer electrospray ion source. Multiple reaction monitoring detection conditions: spray voltage was 2.0 kV; cone voltage was 30 V; desolvation temperature was 400°C; and desolvation gas (N_2_) was 1,000 L/h. Mass spectral scanning conditions: ESI + mode: spray voltage was 3.0 kV; cone voltage was 30 V; desolvation temperature 400°C; and desolvation gas (N_2_) was 1,000 L/h; scanning range: 100–2,000 m/Z; ESI‐mode: spray voltage was 0.5 kV; cone hole voltage was 30 V; desolvent temperature was 400°C; and desolvent gas (N_2_) was 1,000 L/h; scan range: 100–2,000 m/Z.

#### 2.2.2 PPO separation and purification in betel nut kernel

##### Extraction of PPO crude enzyme solution

The crude PPO was extracted using the method described previously by Pan (2006) with some modifications, and the frozen fresh betel nut kernel sample (50 g) was blended in 100 ml of ice‐cold phosphate buffer solution (PBS) (0.1M, pH 6.8 containing 5% PVP) and then homogenized with a Waring Blender (800S). The homogenate was leached at 4°C for 2 hr and filtered with four layers of degreasing gauze. The filtrate was then centrifuged at 10,000 *g* for 20 min. The supernatant was collected as the crude PPO. All the extracting steps were conducted at 4°C.

##### Ammonium sulfate gradation precipitation

Solid ammonium sulfate was added to the crude PPO until 30% of saturation was reached. The solution was then rested at 4°C for 2 hr and then centrifuged at 12,000 *g* for 20 min to collect supernatant. Subsequently, more solid ammonium sulfate was added to the supernatant reaching a final saturation of 70%. The solution was stored overnight at 4°C and next centrifuged at 12,000 *g* for 30 min to achieve precipitated fraction, which was dissolved in a minimum volume of PBS (0.1M, pH 6.8) and then dialyzed overnight against the same buffer to remove salts. The solution was then concentrated by freeze‐drying.

##### DEAE–sepharose fast flow column chromatography

The method was used according to the Mishra et al. (2012) with some modifications. The dialyzed sample (3 ml) was deposited on a DEAE–Sepharose FF column (2 × 25 cm) previously equilibrated with 2–3 times the volume of PBS (0.1M, pH 6.8). The bound proteins were eluted with a linear increasing gradient of NaCl prepared in same buffer (ranging from 0 to 0.5 M) at a flow rate of 1.2 ml/min (3.0 ml/tube). The proteins were monitored at 280 nm using an online UV detector (HD‐3, Nanjing University), and the peak fractions were collected to assay the PPO activity. Fractions having the PPO activity were pooled, and the pooled fraction was dialyzed against the same buffer overnight and concentrated by freeze‐drying.

##### Phenyl–sepharose 6 fast flow column chromatography

The phenyl–Sepharose FF column (2 × 15 cm) was first equilibrated with a solution containing 1.5 M (NH_4_)_2_SO_4_ and then collected eluted solution having the PPO activity on Section 2.2.2.2, and the eluate was lyophilized to enzyme powder, which was then dissolved in 1 ml of 1.5 M (NH_4_)_2_SO_4_ solution and loaded onto the column. Then, we used the above buffer containing 1.5–0.5 M (NH_4_)_2_SO_4_ to perform linear elution from high concentration to low concentration (0.6 ml/min, 3.0 ml/tube), respectively, to determine elution peaks with PPO activity and collected the eluted solution having the PPO activity peak. The purified enzyme solution was freeze‐dried and refrigerated at −20°C used for the next experimental procedure.

##### PPO activity

Each 3 ml reaction mix contained 0.5 M catechol, 0.1 M sodium phosphate buffer, pH 6.8, and 0.2 ml purified enzyme extract solution. The increase in absorbance at 398 nm was monitored. One unit of PPO activity was defined as the amount of enzyme that caused a change in A_398_ of 0.001 U/mL.

#### 2.2.3 Scanning electron microscopy

The betel nut test after washing with distilled water was cut into small pieces of 2 mm, quickly placed in a 2.5% glutaraldehyde solution of pH 6.8, and taken out after fixing at 4°C for 24 hr, and then rinsed passed through a 0.1 mol/L phosphate buffer (pH 7.2), then dehydrate for 15 min in 50%, 70%, 80%, 90%, and 95% ethanol, and dehydrate 100% ethanol for 30 min. After vacuum freeze‐drying for 48 hr, the samples were adhered to the sample stage with a conductive adhesive, the ion sputter coating apparatus was plated with a gold film and placed under a scanning electron microscope (S‐3000N, Japan Hitachi Company) at an acceleration voltage of 10 kV to observe the film.

#### 2.2.4 Browning type identification

##### Preparation of extracts from natural browning products of betel nut kernel

The betel nut was half‐cut and stored at room temperature. After the kernel were browned, the browning part of the surface was cut with a knife, and 20 g was accurately weighed and extracted by ethanol water bath reflux method. The ethanol solution was recovered by rotary evaporation at 70°C, hydrolyzed with sulfuric acid (20%) and then extracted with chloroform in a ratio of 1:1, and chloroform was recovered by rotary evaporation at 36°C to obtain a browning product extract.

##### Full‐band scanning comparison of oxidation products and natural browning products

The purified and dried PPO enzyme powder was dissolved in PBS of pH 6.8, and the three phenolic substances, chlorogenic acid, dopamine, and epicatechin, which are the most abundant in the betel nut, were simulated in vitro according to the peak area ratio under aerobic conditions. The oxidation reaction products and natural browning product extracts were spectrally scanned in the same wavelength range of 200–700 nm ultraviolet–visible spectrophotometer (T6 Series, General Analysis Equipment Co., Ltd), and the spectra obtained by the two scans were compared.

## 3 RESULTS

### 3.1 HPLC chromatographic separation of phenolic compounds in betel nut kernel

**Figure 1:**
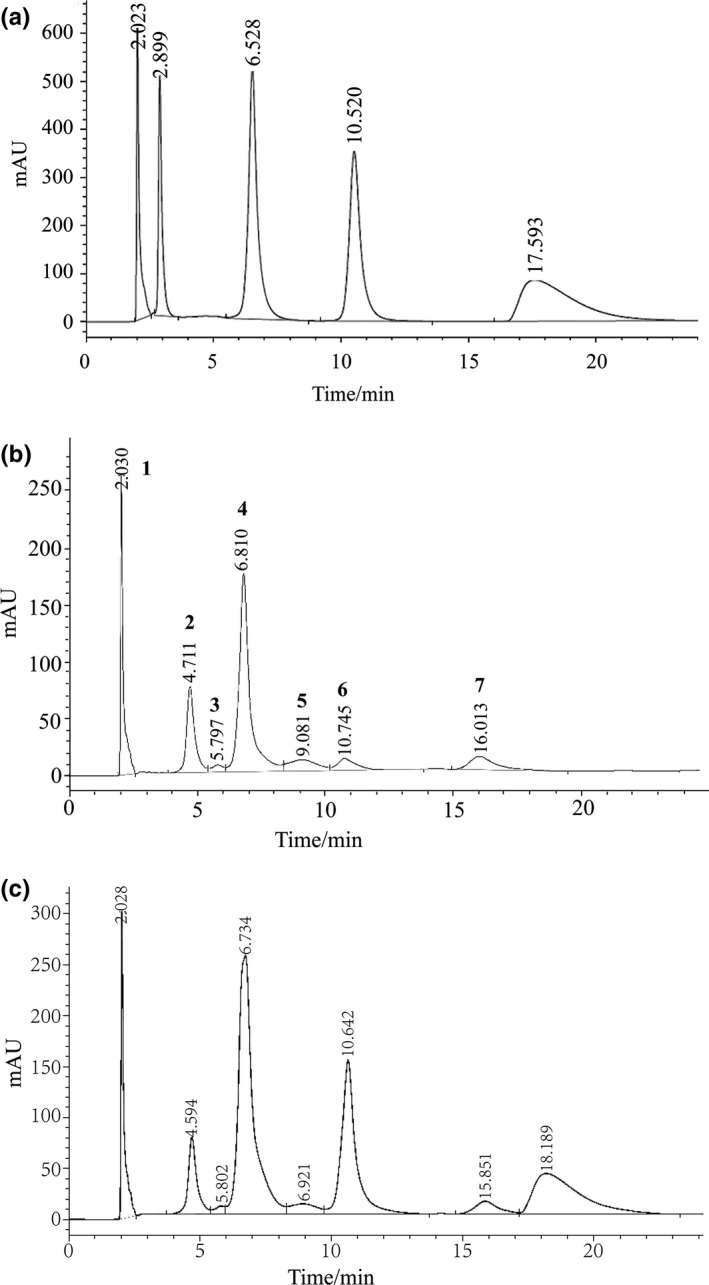
Chromatogram of polyphenol standard (a), chromatogram of methanol extract of betel nut kernel (b), and internal standard chromatogram of methanol extract (c);1–7, Peak sequence number

As shown in Figure 1a, the order of the peaks of the standard samples is dopamine, gallic acid, chlorogenic acid, epicatechin, and catechin. Comparing the retention time of the HPLC chromatogram of the standard sample in Figure 1a, the results showed the methanol extract of phenol compounds in the betel nut kernel: peak 1, peak 4, peak 6, and peak 7 in Figure 1b corresponded with dopamine, chlorogenic acid, epicatechin, and catechin, respectively. And then the above four standard samples were added to the methanol extract of betel nut phenolic compounds to obtain the result of Figure1c, peak 1, peak 4, peak 6, and peak area increased, but peak 7 did not change; however, a new peak appeared, indicating that the corresponding substance of peak 7 is not catechin. Through the comprehensive analysis of Figure 1, it can be confirmed that the peak 1, peak 4, and peak 6 are dopamine, chlorogenic acid, and epicatechin. The results showed that chlorogenic acid, dopamine, and epicatechin exist in the betel nut kernel. For phenolic substances without standard comparison, unknown phenolic substances were detected by HPLC‐MS technique using the multiple reaction detection mode (MRN) of triples rod mass spectrometry (Waters Xevo TQ‐S Micro) and further confirmed the identified phenolic substances.

### 3.2 Mass spectrometry analysis of phenolic compounds in betel nut kernel

By analyzing the relative molecular mass and fragmentation in Table 1, the primary mass spectrometry, secondary mass spectrometry library, and related literature analysis are combined. Based on the three phenolic compounds identified in the liquid phase, the remaining eight phenolic substances were measured, a total of 11 phenolic substances were inferred, and they were dopamine, chlorogenic acid, L‐epicatechin, protocatechuic acid, ferulic acid, ρ‐hydroxycinnamic acid, sinapic acid, quinic acid, procyanidins B_2_, rutin, and hyperoside. According to the peak area normalization method, we found that the content of chlorogenic acid was the highest, followed by dopamine and epicatechin, and other contents were relatively small.

**Table 1:** The mass spectrometry data of phenolic compounds in areca nut kernel

Retention time/min	Relative molecular mass	Peak area	Parent ion	Daughter ion	Determine compounds
2.735	192	48	191	127.1, 108.1, 92.7, 84.9	Quinic acid
3.004	153	289,356	153.9	124, 77, 51, 36	Dopamine
5.801	154	3,495	152.9	109, 80.9	Protocatechuic acid
7.375	354	562,616	353.2	308.9, 191.4, 134.8, 85.1, 28	Chlorogenic acid
10.696	290	90,280	289.1	203, 151, 123, 109	L‐epicatechin
14.45	578	67,467	577	425, 407	Procyanidins B2
18.03	164	108	163	119, 91	p‐Hydroxycinnamic acid
22.391	194	560	193	178, 149, 134	Ferulic acid
23.51	224	460	223	163, 149, 121	Sinapic acid
24.53	610	3,601	609	301.1, 270.9, 178.7, 151	Rutin
26.017	464	2,125	463	300.7, 270.9, 254.9, 30, 30	Hyperoside

### 3.3 Separation and purification of PPO in betel nut kernel

**Figure 2:**
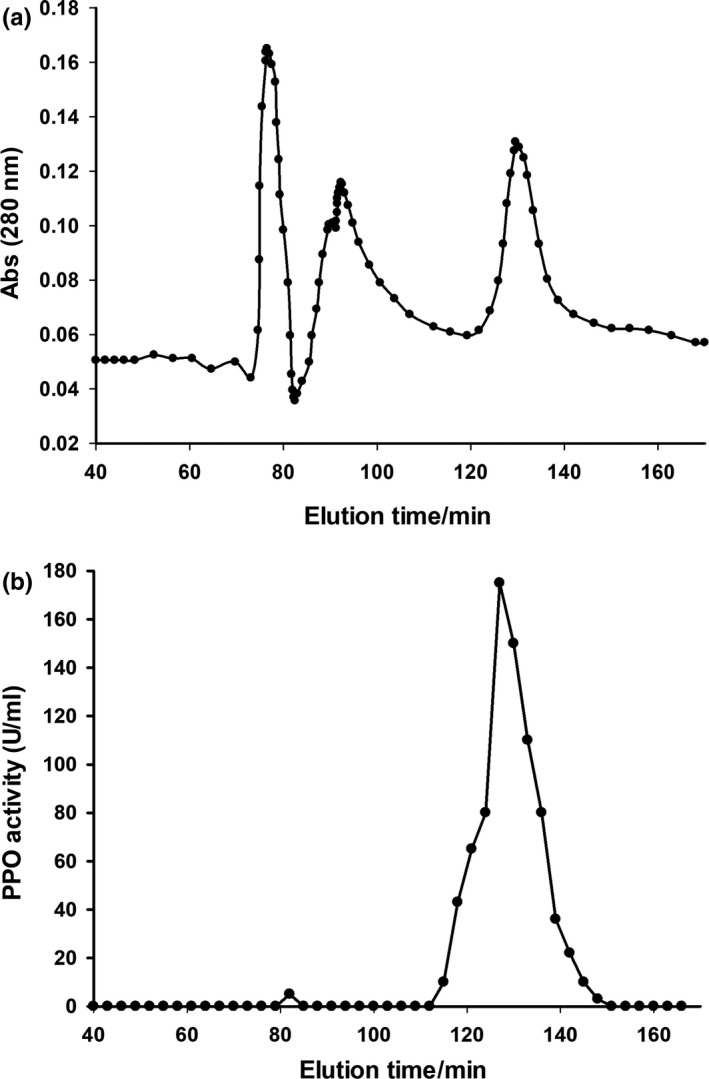
Chromatographic elution profile (a) and enzyme activity peak (b) of PPO from betel nut kernel on DEAE–Sepharose Fast Flow. PPO, polyphenol oxidase

**Figure 3:**
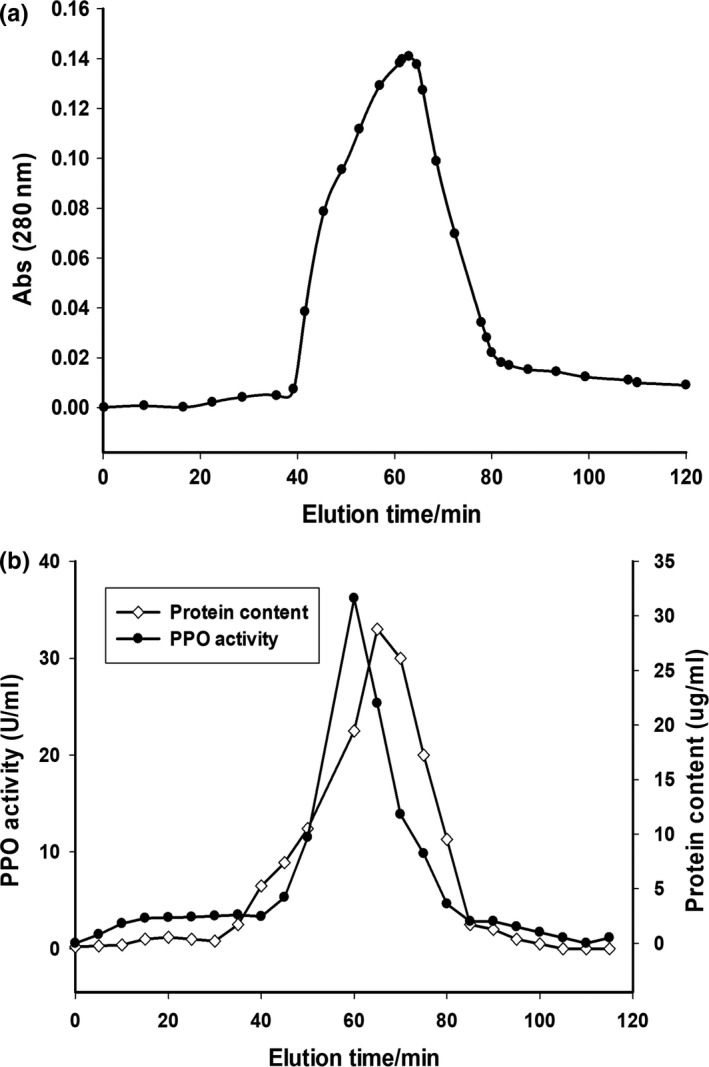
Chromatographic elution profile (a) and enzyme activity peak (b) of PPO from betel nut kernel on Phenyl–Sepharose 6 Fast Flow. PPO, polyphenol oxidase

As can be seen from Figure 2a, three eluting peaks appeared during the elution. Among them, the first two eluting peaks had no PPO enzyme activity, and only the third eluting peak had PPO enzyme activity (Figure 2b), thereby confirming that PPO was in the third eluting peak. Therefore, the enzyme solution of the third eluting peak was collected, lyophilized by dialysis for 24 hr, and subjected to Phenyl–Sepharose 6 Fast Flow column chromatography. As shown in Figure 3, only one elution peak appeared in the elution solution and had high PPO activity, then collected the eluted solution having the PPO activity in Figure 2b and refrigerate it at −20°C used for the next experimental procedure. It was indicated that the enzyme had been separated and purified by DEAE–Sepharose and phenyl–Sepharose column chromatography. So, this result illustrates the presence of PPO in the kernel.

### 3.4 Changes in the structure of betel nut fruits

**Figure 4:**
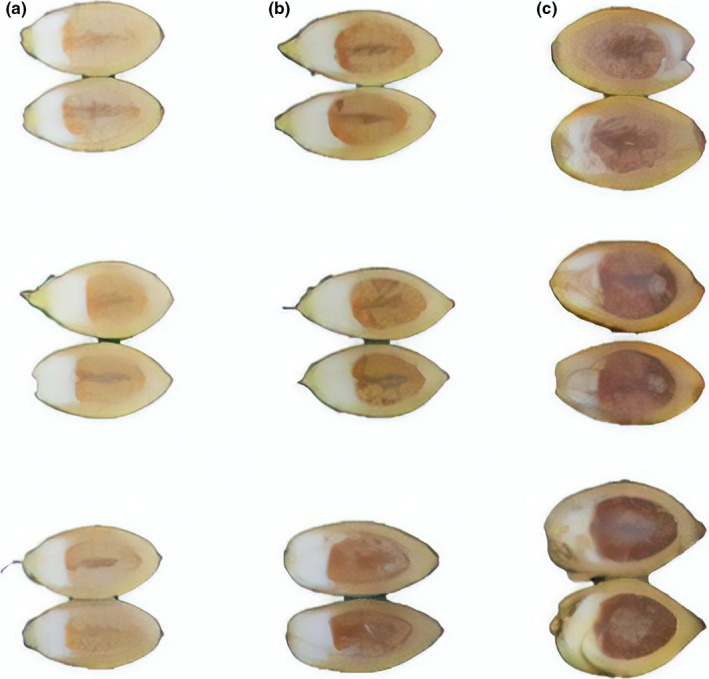
Browning symptom of betel nut kernel at room temperature of three storage time. 0 day (a), 10 days (b), and 20 days (c)

**Figure 5:**
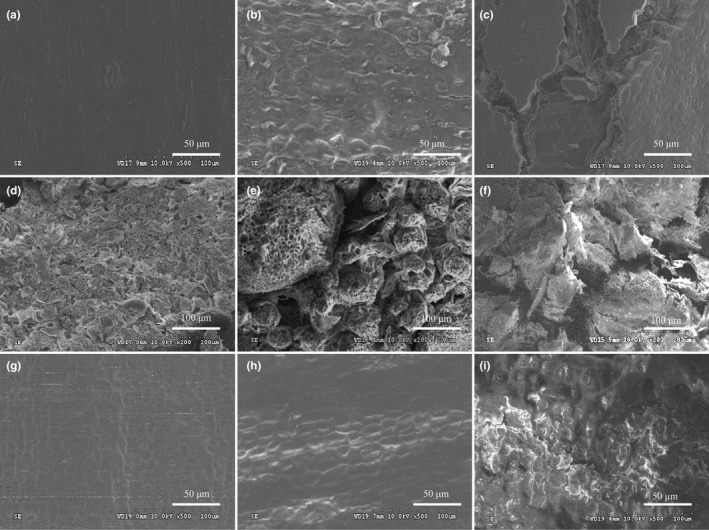
Scanning electron micrographs of peel, flesh, and kernel membrane in areca nut at 25°C. (a–c), peel structure at 0, 10, and 20 days; (d–f), flesh structure at 0, 10, and 20 days; (g–i), kernel membrane structure at 0, 10, and 20 days

Scanning electron microscopy (SEM) shown that the epidermal structure of betel nut fruits was relatively intact at the initial stage of storage (0 day), the surface wax coverage was tight, uniform without fragments, the epidermis had no cracks, and the structure was relatively stable (Figure 5a). At this time, there is no browning in the betel nut kernel (Figure 4). With the prolongation of storage time, in the middle of fruit storage (10 days), the gradual decomposition and coverage of epidermal wax were reduced, and many silky structures and pores appeared in the epidermal keratin membrane, which were scaly or honeycomb‐like (Figure 5b). At the end of the period (20 days), the browning of the kernel has become quite serious (Figure 4). Simultaneously, the epidermis was damaged and picked up, and the surface was split into lip‐like protrusions to form lenticels, and the length and width of the sulcus were increased (Figure 5c). It is obvious that the destruction of the epidermal tissue integrity of the betel nut fruit will inevitably contribute to the gas exchange inside and outside the organization, including the entry of O_2_. As shown in Figure 5d, in the initial stage of storage, the cross‐section of the betel nut fruit is relatively flat and uniform, the tissue arrangement is neat and orderly, and the pulp tissue structure is relatively complete with no interstitial space. By the middle of storage, the arrangement of pulp tissue became disordered, the joints between tissues gradually relaxed, and the joints gradually decreased and separated from other tissues (Figure 5e). By the end of storage, the interstitial space of the pulp section increased and the structural damage was particularly serious (Figure 5f). This structural change will inevitably contribute to the exchange of the gas, including O_2_ entering the interior of the tissue. As shown in Figure 5g, in the initial stage of storage, the betel nut fruit has a complete membrane structure with a smooth surface and no wrinkles. As the storage time prolonged, the bio‐cellular membrane of the kernel contracted, and the membrane gradually appeared cracks and breakage, which destroyed the membrane permeability (Figure 5h,i). The fruit kernel membrane is the last natural barrier of O_2_ into the kernel tissue. The destruction of its structure will inevitably lead to the opening of the O_2_ into the final channel and finally provide the necessary condition for the enzymatic browning of the kernel—O_2_.

### 3.5 Comparison of spectral scanning results between the reaction products of exogenous phenolic compounds and purified PPO and natural browning extracts of betel nut kernel

**Figure 6:**
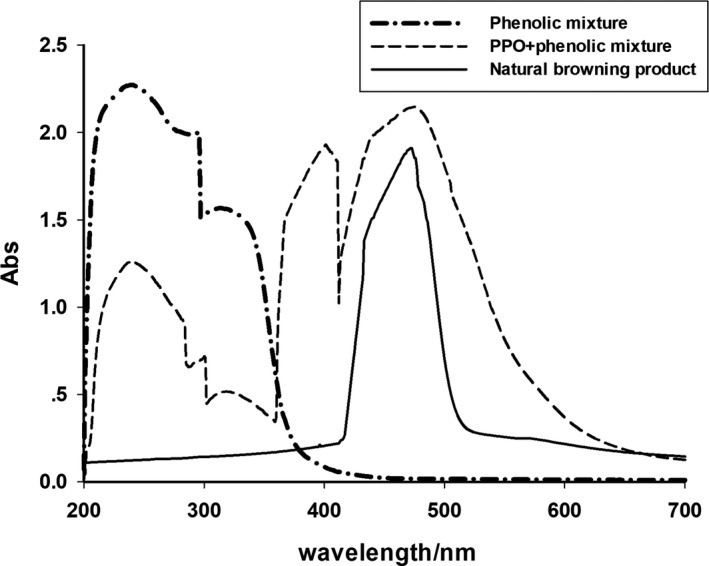
Spectral scans of exogenous phenolic compounds and purified PPO reaction products and natural browning extracts. PPO, polyphenol oxidase

As shown in Figure 6, the absorption spectra of chlorogenic acid, dopamine, and epicatechin were 239, 296, and 313 nm, respectively. After the addition of purified PPO, the absorption peaks of the three phenolic compounds decreased significantly, and at the same time, a maximum absorption range appeared at 475 nm. The new maximum absorption peak, which is similar to the absorption spectrum of the betel nut browning extract, extracted under natural browning conditions. It shows that the essence of browning of betel nut kernel is an enzymatic browning, which is the result of the browning reaction of endogenous substrate—phenolic substances catalyzed by its endogenous PPO.

## 4 DISCUSSION

Browning is one of the factors affecting the quality of fruits after harvest. For fruits, postharvest browning is mainly caused by enzymatic browning (Adams & Brown, 2007; He & Luo, 2007). However, the occurrence of enzymatic browning of fruits generally occurs on the surface of the fruit or on the injured part (Toivonen & Brummell, 2008). There is a little chance of browning inside the fruit. This may be mainly due to the difficulty of oxygen entering the interior of the fruit. However, there are also a few fruits, such as pineapple and pear fruit, which will have an internal black heart after harvest. From the current research, the occurrence of these browning symptoms is related to enzymatic browning (Avallone, Guiraud, & Brillouet, 2003; Franck et al., 2007; Koushesh & Moradi, 2016; Luengwilai et al., 2016; Zhou, Dahler, Sjr, & Rbh, 2003). For betel nut kernel, this study shows that during storage there are more types of phenolic compounds in the betel nut kernel. Among them, chlorogenic acid, dopamine, and L‐epicatechins are all common substances that can cause enzymatic browning. These phenols have proven to be substrates for enzymatic browning on many fruits. As in the case of pears, browning occurs on the pears due to the enzymatic oxidation of the main phenolic substrates chlorogenic acid and L‐epicatechin (Amiot, Tacchini, & Aubert, 1995). Evidence has been presented that L‐epicatechin isolated from litchi is the direct substrate oxidized by isolated pericarp PPO (Liu, Cao, Xu, & Zhang, 2010; Sun, Jiang, & Wei, 2006). Dopamine is the main phenolic browning substrate in banana fruit (Romphophak, Siriphanich, & Ueda, 2005) Therefore, the presence of these phenolic substances provides a basis for the enzymatic browning of betel nut kernel. At the same time, this study also isolated and purified the key enzyme of enzymatic browning‐PPO from the betel nut kernel, which further provides possibility of enzymatic browning of betel nut kernel. In many fruits with enzymatic browning, there is the presence of PPO activity, including the black heart disease of pineapple and pear. The research about pineapple found that theo‐quinones intermediates produced by pineapple PPO act on the phenolic compounds and are then converted to the polymers responsible for the internal enzymatic browning (Hu, Li, Dong, & Chen, 2012; Stewart, Sawyer, & Bucheli, 2001). And the chlorogenic acid was the most suitable substrate for PPO in enzymatic browning of “Laiyang” avocadoes and “Yali” pears pulp (Zhou et al., 2003; Zou, Qiu, Zhao, & Jiang, 2014), which further proved the role of PPO in fruit enzymatic browning.

During storage, the peel wax has the function of preventing the gas component from penetrating the fruit (Huo & Li, 1992; Li, Li, Fand & Li, 2016). It is known from the research that with the prolongation of the storage time of the betel nut, the wax covered on the surface of the peel is gradually decomposed, and the length and width of the stratum corneum increased. At the same time, the lignification of the pulp tissue is aggravated (Singh, Rastogi, Dwivedi, 2010), which causes an increase in the interstitial space of the pulp tissue. Subsequently, the destruction of kernel membrane will lead to open the last natural barrier of O_2_ into the kernel tissue. These structural changes will lead to a significant increase in gas exchange inside and outside the tissue, which provides the necessary condition for the browning of the kernel–O_2_ and promote biological oxidation processes inside the fruit, thereby promoting the enzymatic browning of the kernel (Huo & Li, 1992; Li et al., 2016). Wax coating treatment can reduce the black heart of pineapple (Hu et al., 2012) and betel nut (Li et al., 2017) by maintaining the structural integrity of the peel to further verify the effect of structural changes on gas permeability. In summary, the destruction of the betel nut structure causes the possibility of oxygen entering the kernel, with the increase of storage time, the damage degree of the structure of the betel nut is positively correlated with the browning of kernel, the more serious the structure of betel nut was destroyed, the more O_2_ entered the kernel, and the more serious the browning was.

Finally, the endogenous PPO of betel nut kernel and its mixture of chlorogenic acid, dopamine, and L‐epicatechin interact under aerobic conditions to produce a browning reaction, forming a browning products, and it was found that the absorption spectrum of the browning product was basically same as the browning sample of betel nut kernel, indicating that the essence of betel nut kernel browning is an enzymatic browning, which is the result of endogenous PPO catalyzing the enzymatic browning reaction of its substrates (phenolic compounds). This is the same as the browning nature of pineapple and pears.

Emphasisly, enzymatic browning does not occur in intact plant cells since phenolic compounds in cell vacuoles are separated from the PPO that is located in the thylakoid lumen of the plant cells (He & Luo, 2007; Ioannou & Ghoul, 2013; Astrid‐Kim et al., 2011). So how to destroy distribution of cell regionalization and cause a contact of PPO with phenolic substrate to induce enzymatic browning reaction during the storage of the betel nut kernel needs to be further researched.

## 5 CONCLUSIONS

In this research, there are 11 phenolic compositions of betel nut kernel to be identified by quantitative analysis of HPLC‐MS. These phenolic compounds included chlorogenic acid, dopamine, L‐epicatechin, protocatechuic acid, ferulic acid, p‐hydroxycinnamic acid, sinapic acid, quinic acid, procyanidins B_2_, rutin, and hyperoside. Among them, the content of chlorogenic acid, dopamine, and epicatechin are relatively high. At the same time, chromatographically pure PPO was isolated from the nuts. On this basis, SEM observation found that the cuticle of the peel was honeycomb‐shaped, and the wax was gradually decomposed; the lignification of the pulp was aggravated, and the interstitial space was increased; and the crack of the kernel film was broken and damaged with the prolonged storage time. These changes help to increase gas exchange within and outside the organization, including the entry of O_2_. Finally, the oxidized products obtained by the simulated purification of PPO and chlorogenic acid, dopamine, and epicatechin under aerobic conditions in vitro were compared with the products extracted from brown betel nut, and the two were identical. The absorption spectrum indicates that browning of betel nuts is an enzymatic browning caused by the reaction of PPO and phenolic substrates.

## CONFLICT OF INTEREST

The authors declare that they do not have any conflict of interest.

## ETHICAL STATEMENT

This study does not involve any human testing.
